# X-ray sequence and crystal structure of luffaculin 1, a novel type 1 ribosome-inactivating protein

**DOI:** 10.1186/1472-6807-7-29

**Published:** 2007-04-30

**Authors:** Xiaomin Hou, Minghuang Chen, Liqing Chen, Edward J Meehan, Jieming Xie, Mingdong Huang

**Affiliations:** 1 State Key Laboratory of Structural Chemistry, Fujian Institute of Research on the Structure of Matter, The Chinese Academy of Sciences, 155 Yang Qiao Xi Lu, Fuzhou, Fujian, 350002, China; 2 Graduate School of Chinese Academy of Sciences, The Chinese Academy of Sciences, Beijing 10039, China; 3 Laboratory for Structural Biology, Department of Chemistry, Graduate Programs of Biotechnology, Chemistry and Materials Science, University of Alabama in Huntsville, Huntsville, AL 35899, USA; 4 Fujian Medical University, Fuzhou 350004, China

## Abstract

**Background:**

Protein sequence can be obtained through Edman degradation, mass spectrometry, or cDNA sequencing. High resolution X-ray crystallography can also be used to derive protein sequence information, but faces the difficulty in distinguishing the Asp/Asn, Glu/Gln, and Val/Thr pairs. Luffaculin 1 is a new type 1 ribosome-inactivating protein (RIP) isolated from the seeds of *Luffa acutangula*. Besides rRNA N-glycosidase activity, luffaculin 1 also demonstrates activities including inhibiting tumor cells' proliferation and inducing tumor cells' differentiation.

**Results:**

The crystal structure of luffaculin 1 was determined at 1.4 Å resolution. Its amino-acid sequence was derived from this high resolution structure using the following criteria: 1) high resolution electron density; 2) comparison of electron density between two molecules that exist in the same crystal; 3) evaluation of the chemical environment of residues to break down the sequence assignment ambiguity in residue pairs Glu/Gln, Asp/Asn, and Val/Thr; 4) comparison with sequences of the homologous proteins. Using the criteria 1 and 2, 66% of the residues can be assigned. By incorporating with criterion 3, 86% of the residues were assigned, suggesting the effectiveness of chemical environment evaluation in breaking down residue ambiguity. In total, 94% of the luffaculin 1 sequence was assigned with high confidence using this improved X-ray sequencing strategy. Two N-acetylglucosamine moieties, linked respectively to the residues Asn77 and Asn84, can be identified in the structure. Residues Tyr70, Tyr110, Glu159 and Arg162 define the active site of luffaculin 1 as an RNA N-glycosidase.

**Conclusion:**

X-ray sequencing method can be effective to derive sequence information of proteins. The evaluation of the chemical environment of residues is a useful method to break down the assignment ambiguity in Glu/Gln, Asp/Asn, and Val/Thr pairs. The sequence and the crystal structure confirm that luffaculin 1 is a new type 1 RIP.

## Background

The amino-acid sequence is the most basic but critical information for proteins. The primary structure of the protein can be obtained through Edman degradation, mass spectrometry or cDNA method. These methods, especially cDNA method, have been applied widely, but they still have their own limitations [1]. Edman degradation is expensive and cannot deal with cases where the N-terminal amino-acid of protein is blocked. cDNA sequencing is the most popular sequencing method nowadays, but it cannot identify the amino-acid post translational modifications. Mass spectrometry has made dramatic advance in the last decade in its technology [2], but still has difficulties to give the full-length sequence and to distinguish the residue pair Ile/Leu. X-ray sequencing method, based on electron density, is another method to determine the protein sequence. Although this method has limited usage, it is a useful addition to the sequencing methods in some cases, e.g., where cDNA is not readily available. This method has been used to determine the sequence of PAP-S_aci _[3] and trichomaglin [4]. A major problem of this method is the difficulty to distinguish residue pairs Asp/Asn, Glu/Gln, and Val/Thr. Moreover, weak electron density of some residues located at the molecular surface also gives rise to uncertainty for the X-ray sequence analysis. Here, we demonstrate that the evaluation of the chemical environments of these pairs can help to break down such ambiguity and 86% (see Table 2) of the amino acids were assigned with confidence on the basis of the electron density and the chemical environment evaluation of the residues.

The ribosome-inactivating proteins (RIPs) are RNA N-glycosidases [5,6] that inactivate ribosome by cleaving a single N-C glycosidic bond between adenine and ribose at A4324 in the 28S eukaryotic mammalian rRNA or at A2660 in the 23S *Escherichia coli *rRNA. The cleaved N-C glycosidic bond is located in a loop containing a GAGA sequence and highly conserved in rRNAs from bacteria, plants and animals. The removal of one adenine from rRNA by RIPs prevents the binding of elongation factor II (EF-2) to the 60S subunit, resulting in the termination of protein translation. In E. coli, the cleavage by RIP affects the combination of EF-Tu and EF-G. Both EF-G and EF-Tu protect bases in the universally conserved loop around position 2660 of 23S rRNA. This loop is also the site of action of cytotoxins that alter the structure of a region of rRNA that interacts with EF-Tu and EF-G and thus abolish protein synthesis. RIPs from plants can be classified into three types based on the structure of the genes and mature proteins [7]. Type 1 RIPs, such as trichosanthin [8], bryodin [9], α, β-momorcharin [10,11], luffin a and b [12] and cucurmosin [13], have alkaline isoelectric points and molecular weights ranging from 26 to 31 kDa. They typically contain a single polypeptide chain and have the potent ability to inhibit protein synthesis in the cell free system but are relatively non-toxic to the intact cells. Type 2 RIPs, such as ricin [14] and abrin [15], consist of two chains, chain A and chain B, linked by disulfide bridges. The A chain is homologous to type 1 RIPs and possesses the ribosome-inactivating activity; the B chain, containing a lectin domain, binds to galactosyl-terminated receptors on the target cell surface, facilitating the entry of the A chain into the cytoplasm of the cell. Thus, some, but not all, type 2 RIPs are more potent toxin than type 1 RIPs because type 1 RIPs have difficulty in entering into cells. Type 3 RIP includes JIP60 [16] (jasmonate-induced protein) from maize, which consists of an N-terminal domain similar to type 1 RIPs and an unrelated C-terminal domain of unknown function. Most RIPs are glycoproteins, with varying amount and type of sugars.

RIPs have received wide attentions due to their potential therapeutic applications in medicine and transgenic reagents in agriculture. In medicine, they have been found to possess various pharmacological activities including abortifacient [17], antifungal [18], anti-tumor [19,20], antivirus and HIV-1 integrase inhibitory activity [21,22]. Plants transfected with RIP genes exhibit broad-spectrum resistance to viral and fungal infection [23,24] in the plant defense system.

We identified a new type 1 RIP, luffaculin 1 [25]. It is a basic protein with a pI of 8.86 by IEF analysis and has a molecular mass of about 28 kDa based on the mobility on SDS-PAGE. Luffaculin 1 not only possesses rRNA N-glycosidase activity as expected [26], but also inhibits proliferation of tumor cells, induces apoptosis [27] and differentiation on tumor cells [28].

Here, we report the high resolution (1.4 Å) crystal structure of luffaculin 1 and the protein sequence derived from this crystal structure. The structural comparison with other RIPs provides a structural basis to understand their possible biological activity. The amino-acid sequence of luffaculin 1 has not been determined by the traditional cDNA method. We demonstrated that the primary structure of luffaculin 1 can be derived with a high degree of confidence from the high-resolution electron density. The existence of two independent luffaculin 1 molecules in the asymmetric unit allows the cross-validation of this X-ray sequence, further increasing the reliability of the sequence assignment.

## Results and discussion

### Quality of the model

The crystals of luffaculin 1 belong to space group P1 and diffract to 1.4 Å resolution with synchrotron X-ray radiation. A high quality data set was collected to a completeness of 86.7% and redundancy of 1.9, yielding an Rmerge of 0.03 (Table 1). The current model of luffaculin 1, containing two molecules in the asymmetric unit, was refined to an R factor of 0.213 and R_free _of 0.232. Most residues in the model fit the electron density quite well, except for some residues in loop regions that do not have good quality of electron density. Residues 28, 206, 215–220 of molecule B were omitted from the final model due to lack of the electron density. The quality of the stereochemistry of the final protein structure was analyzed by PROCHECK package [29]. The root mean square deviations (rmsd) of bond length and bond angles are 0.007 Å and 1.153°, respectively. The Ramachandran plot shows 91.3% of the residues in the most favored region and 7.6% in the additional allowed region. Residues Asn 77 of molecules A and B, which are linked to an N-acetylglucosamine, respectively, lie just outside the generously allowed region. Residue Tyr 141 of the molecule B, located in a turn connecting α6 helix and α7 helix, and residue Asn 235 of the molecule A, located in a turn connecting α9 helix and α10 helix, lie in the generously allowed region. Data collection and refinement statistics are summarized in Table 1.

### Structure description

**Figure 1:**
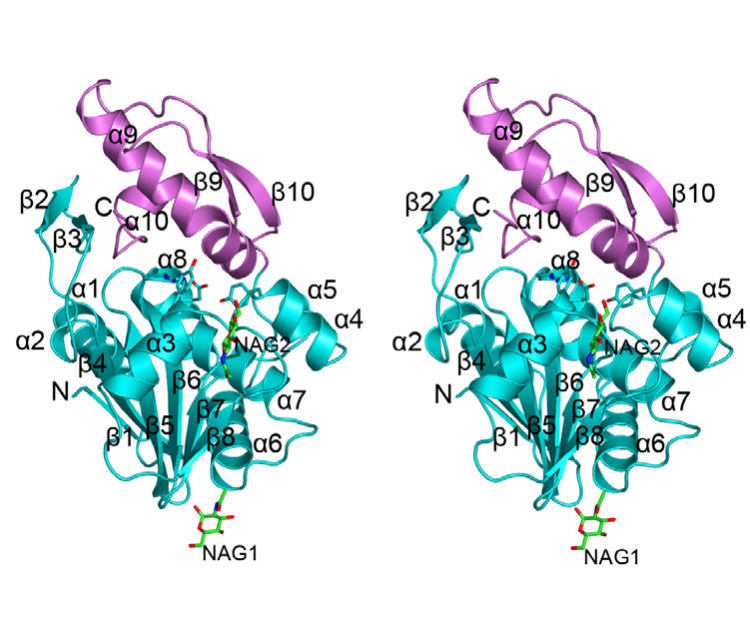
**Overall structure of luffaculin 1 in stereo representation**. The two domains at the N- and C-terminal were colored in cyan and violet, respectively. Stick representations are residues Tyr70, Tyr110, Glu159 and Arg162 in the active site, and the two N-acetylglucosamines that are each covalently linked to Asn 77 and 84, respectively. All figures except for Fig. 4 were prepared using Pymol [46].

Fig. 1 shows a ribbon representation of luffaculin 1. The structure of luffaculin 1 contains two domains: a large N-terminal domain composed of eight α-helices and eight β-strands, and a smaller C-terminal domain consisting of two α-helices (α9 and α10) and two β-strands (β9 and β10). The secondary structure of luffaculin 1 is typical of type 1 RIPs: six β-strands of N-terminal domain (β1, β4, β5, β6, β7 and β8) form a mixed β-sheet. Eight helices of N-terminal have canonical geometry [30] and enclose the active site cleft. Helices α1 and α3 are part of the crossover connections between the parallel strands of the β-sheet. Helices α7 and α8 are contiguous in sequence and a single residue (Phe 163) assumes a non-helical dihedral conformation, introducing a bend between the two helices. Two β-strands of C-terminal domain (β9 and β10) are connected by a loop whose length varied among different RIPs.

**Figure 2:**
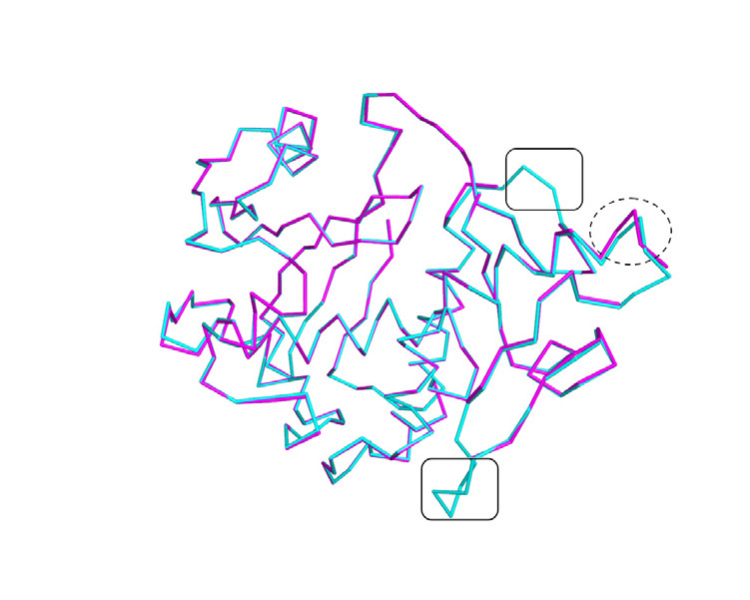
**Superposition of molecule A (cyan) and B (magenta) of luffaculin 1**. The slight deviation is indicated in a dashed circle. Residues not present in the molecule B are outlined in the boxes.

The crystals of luffaculin 1 contain two enzyme molecules (A and B) in the asymmetric unit. A comparison of molecules A and B shows that the overall structures of these two molecules are almost identical (Fig. 2) with rmsd of 0.181 Å for 221 Cα atoms. Some deviation between two molecules occurs at the terminal of the α9-helix that is involved in the crystal packing.

The electron density of luffaculin 1 clearly indicated the existence of two well-defined N-acetylglucosamines (NAGs), each covalently linked to an Asn residue at positions 77 and 84, respectively. Both N-acetylglucomamines protrude from the molecular surface and do not have extensive interaction with the protein. These saccharide moieties of luffaculin 1 are also distant from the active site (Fig. 1), suggesting that these saccharide moieties may not involve in the enzymatic activity. The glycosylation in protein has been recognized to play important roles in various functions, including protein folding in the endoplasmic reticulum, transport and secretion, anchoring of proteins to target sites, protection from protease, and increased protein conformational stability [31,32].

### X-ray sequence of luffaculin 1

**Figure 3:**
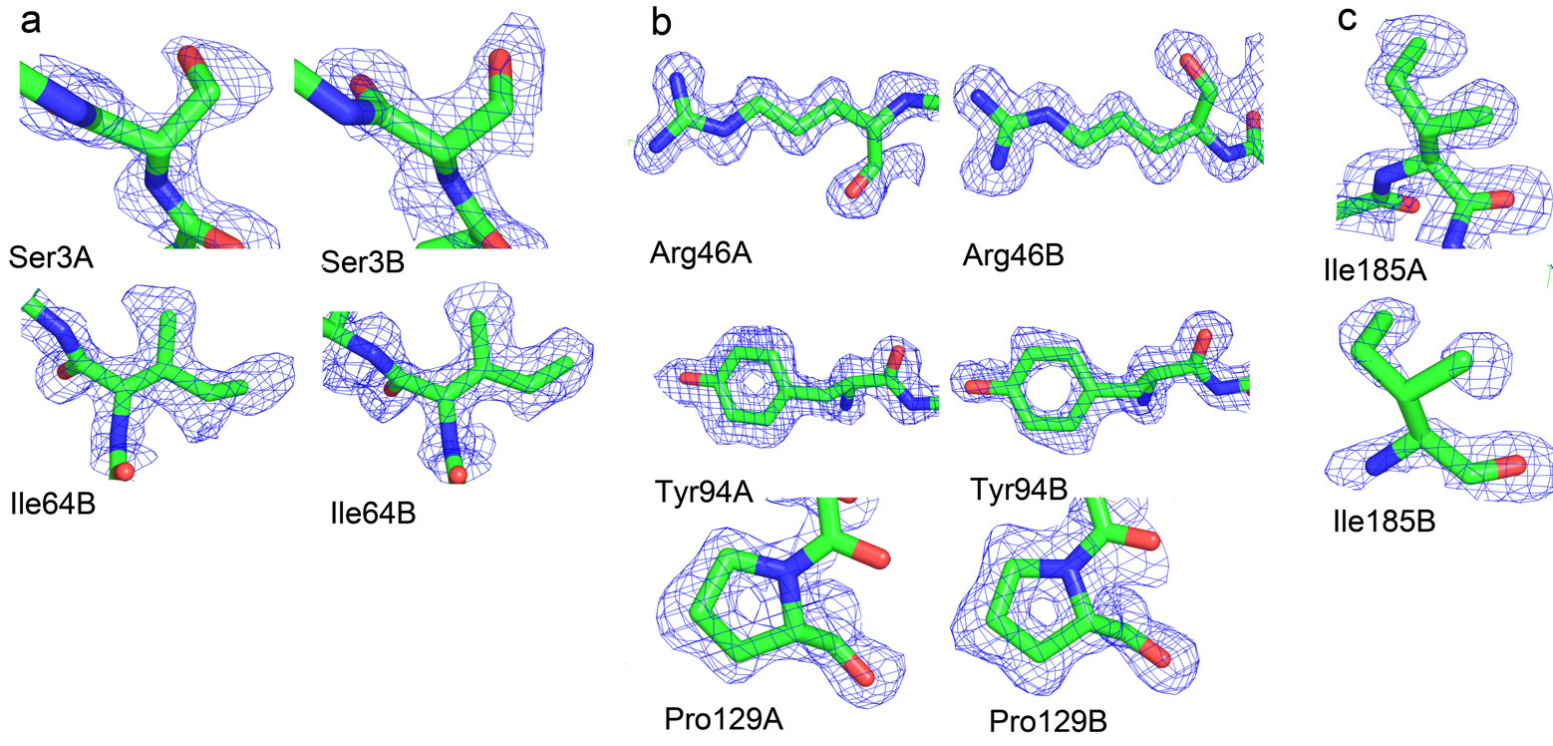
**The presence of two molecules in the asymmetric unit facilitates sequence identification**. (a) shows residues 3 and 64 in molecule A and B of luffaculin 1 contoured at 2σ and these residues are different from luffin a. (b) shows residues 46, 94 and 129 with clear electron density in molecule A and B contoured at 2σ. (c) shows residue 185 with weak electron density in molecule B can be clearly recognized as Ile in the electron density of molecule A.

The sequence of luffaculin 1 was unknown. Only the first five residues in N-terminal region were determined to be DVSFS by N-terminal sequencing. Both luffaculin 1 and luffin a belong to the same genus (Luffa) but different species in the Cucurbitaceae family, thus they are expected to share high sequence homology. For structural determination of luffaculin 1, we used luffin a to build a homology model for molecular replacement method. Although the sequence of luffaculin 1 is not yet known, the high resolution of electron density, combined with the known sequence of homolog luffin a, has allowed us to undertake an 'X-ray sequencing' method with a high degree of confidence. For this purpose, we used annealed composite and σ-weighted 2Fo-Fc omit maps [33] throughout this work to reduce model bias. The electron density allowed us to identify differences between luffaculin 1 and its molecular replacement model, luffin a. For example, residues 3 and 64 were reported as Arg and Val in luffin a, whereas in luffaculin 1 these residues were recognized unambiguously as Ser and Ile, respectively (Fig. 3a).

**Figure 4:**
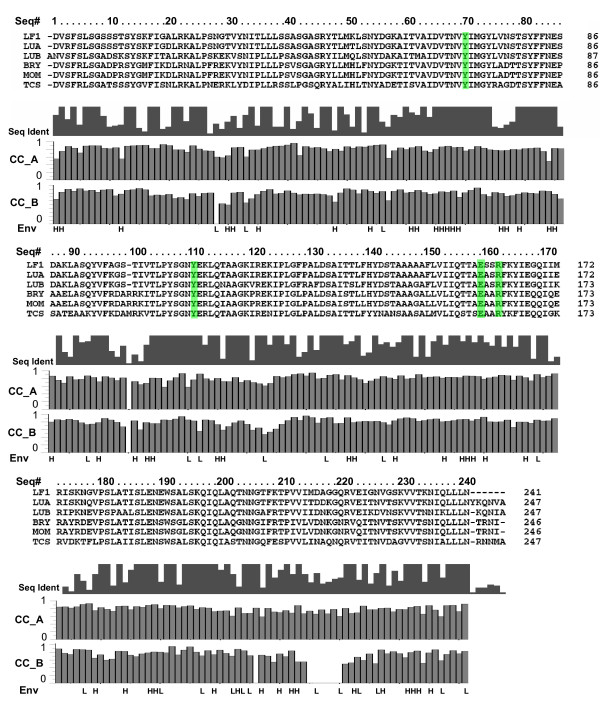
**X-ray sequence of luffaculin 1 and multiple sequence alignments**. These alignments are of luffaculin 1 (LF1), luffin a (LUA), luffin b (LUB), α-momorcharin (MOM), trichosanthin (TCS) and bryodin (BRY). Highlighted residues are the active site residues. Panel Seq Ident indicates the sequence homology calculated by program clustalx 1.83. Panel CC_A and CC_B are the real space fit (RS fit, calculated by program O ranging from 0 to 1) of the residues between current luffaculin 1 and composite omit map of luffaculin 1. Panel Env represents the evaluation of chemical environment on residue pairs (Glu/Gln, Asp/Asn, Val/Thr) that cannot be distinguished by X-ray crystallography. H/L stands for high/low confidence in breaking down this ambiguity based on its chemical environment (hydrophobic interactions, hydrogen bonds and salt bridges).

Fig. 4 shows the final X-ray sequence of luffaculin 1, its alignment with other RIPs, the correlation coefficient (real space fit [34]) between the electron density and the assigned sequence, and the evaluation of chemical environments (hydrophobic interactions, hydrogen bonds and salt bridges) of the residues that cannot be distinguished by electron density (i.e. Asp/Asn, Glu/Gln, and Val/Thr pairs). The reliability of this X-ray sequence assignment is summarized in Table 2.

Based on the electron density, majority of the residues can be assigned unambiguously. Some examples are illustrated in Fig. 3b for residues 46, 94 and 129. The two protein molecules (A and B) in the crystal should have the identical amino-acid sequence. This redundancy provides an additional level of validation on the electron-density-based sequence assignment (Fig. 3a and 3b). In the case that electron density is disordered or weak in one molecule, the electron density of the other molecule allows the identification of the sequence (9 residues are in such case, see Table 2 and Fig. 3c). Fig. 3c shows an example where residue 185 has weak electron density in molecule B but can be clearly identified as Ile in molecule A. 66% of residues (160 out of a total of 241 residues, see Table 2) of luffaculin 1 can be identified with confidence purely based on the high resolution electron density. Similar to our results, a previous study [4] suggested that 60% of residues can be identified reliably based on electron density.

**Figure 5:**
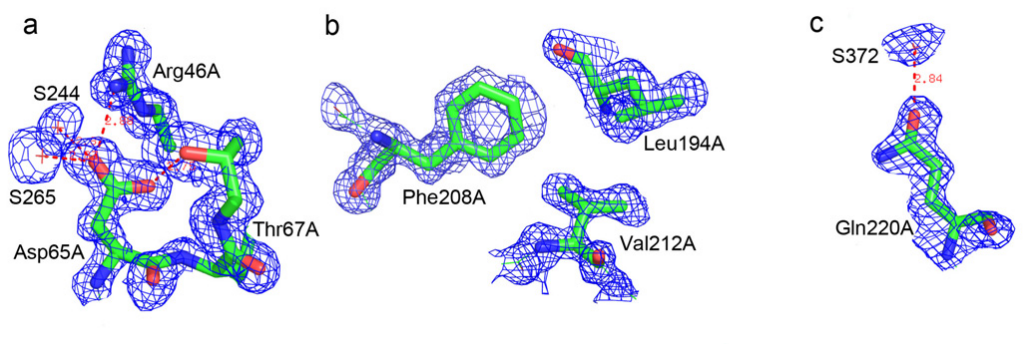
**The electron density of Asp65, Val212 and Gln220 of molecule A**. This map (2Fo-Fc composite omit map) is contoured at 1σ. Hydrogen bonds are denoted by dashed lines with numbers denoting the respective distances in the unit of Å. (a) shows that Asp65 of molecule A has hydrogen bond interaction with Thr67 and salt bridge with Arg46 besides interaction with water molecules S244 and S265. (b) shows that Val212 of molecule A resides in a hydrophobic environment. (c) shows that Gln220 of molecule A has no interaction with other residues except the hydrogen bond with the symmetry related water molecule S327.

An inherent problem of X-ray sequencing is that some residue pairs (Glu/Gln, Asp/Asn, and Val/Thr) can not be distinguished completely based purely on electron density. This is due to the facts that the numbers of electrons in outer shell of carbon, nitrogen, and oxygen are not too much different, and thus cannot be distinguished by X-ray diffraction of protein crystals at a resolution that is usually far less than atomic resolution. In order to solve this problem, we used the information of 1) chemical environment evaluation of amino-acids (hydrophobic interactions, hydrogen bonds and salt bridges); 2) sequence comparison with other RIPs. Information of the glycosylation on Asn residues also facilitates to break down Asp/Asn ambiguity as were the cases for residues Asn77 and Asn84, where carbohydrate moieties were clearly identified from the electron density. Fig. 5a shows that Asp65A has a chemical environment perfect for an Asp, but less possible for an Asn because only an Asp, but not an Asn, can form salt bridge with Arg46. Moreover, Asp65 is quite conserved (Fig. 4) among all RIPs. Four additional residue pairs (Asp87, Glu159, Glu167 and Glu188) were found to form salt bridges with other residues, leading to their convincing assignment. Inspection of hydrophobic environment can also assist to break down Val/Thr ambiguity. Fig. 5b shows an example where the electron density votes for either a Val or a Thr, but the perfect hydrophobic environment makes it less possible for a Thr at this position. This evaluation of chemical environment of residues has increased the assigned residues from 160 (66%) to 208 (86%, see Table 2). It is not surprising that there are still a few residues (marked by asterisks in Table 2) that cannot be distinguished based on their chemical environment. Fig. 5c shows that Gln220 of molecule A has no interaction with any other residues except for a hydrogen bond with a water molecule (S327). The Gln220 in the molecule B is not observed in the electron density, so we cannot conclude A220 as a Glu or Gln. It was reported [3] that the side chain of Ser can distribute among two conformations and may thus look like a Thr residue, albeit at weaker electron density. In our case, no such ambiguity was found.

Four residues (109, 111, 179 and 223) have weak electron density in both molecule A and molecule B (Table 2), and thus cannot be assigned based on electron density. All these four residues are located either at loop region or at the surface of the molecules. However, these four residues are highly conserved among various RIPs (Fig. 4), and are thus tentatively assigned according to the sequences of their homologous proteins. Residues (29, 97, 217, 218, 225 and 228) do not have enough electron density of side chains in both molecules and are not conserved in RIP sequences, and thus cannot be assigned in this study. They are currently tentatively assigned as Ala or Gly.

In summary, the evaluation of chemical environment greatly facilitates to break down the ambiguity (Table 2): 32 out of a total of 36 Val/Thr pairs and 16 out of a total of 38 Asp/Asn and Glu/Gln pairs were assigned by this method. By using electron density and evaluation of chemical environment, 86% of residues were assigned with confidence. Assignment based on sequence comparison, although not absolutely reliable, further increases the number of the identified residues to 227 (94% of a total of 241 residues).

X-ray sequencing method has been successfully used to determine the amino-acid sequence of PAP-S_aci _[3], a Pokeweed antiviral protein, and trichomaglin [4]. The sequence of PAP-S_aci _was obtained from the exceptional quality of the electron density at 1.7 Å resolution, combined with the known sequence of the two PAP-S isoforms. The authors claimed that almost all amino-acid side chains were identified with a high degree of certainty (with the exception of Asp/Asn and Glu/Gln ambiguities). The X-ray sequence of trichomaglin was obtained by combining those derived from electron density at 2.2 Å resolution with the partial sequence information from mass spectroscopic analysis and the experimentally determined N-terminal sequence. 60% of the X-ray sequence was thus demonstrated to be highly reliable. In this paper we got the X-ray sequence of luffaculin 1 based on the high resolution (1.4 Å) electron density, cross-validated by the second molecule in the asymmetric unit of the crystals, and combined with the evaluation of the chemical environment of selected residues.

### Comparison with other RIPs and the active site

Fig. 4 shows the structure-based alignment of luffaculin 1 with selected type 1 RIPs, including luffaculin 1, luffin a, luffin b, α-momorcharin, trichosanthin and bryodin. The amino-acid sequence of luffaculin 1 shows high degree of sequence identities to other RIPs: 94% for luffin a, 83% for luffin b, 73% for α-momorcharin, 64% for bryodin, and 63% for trichosanthin, respectively. The active site is the most conserved region at both sequence and structure level as observed. This indicates that the function and the enzymatic mechanisms of luffaculin 1 are probably the same as other RIPs [35].

**Figure 6:**
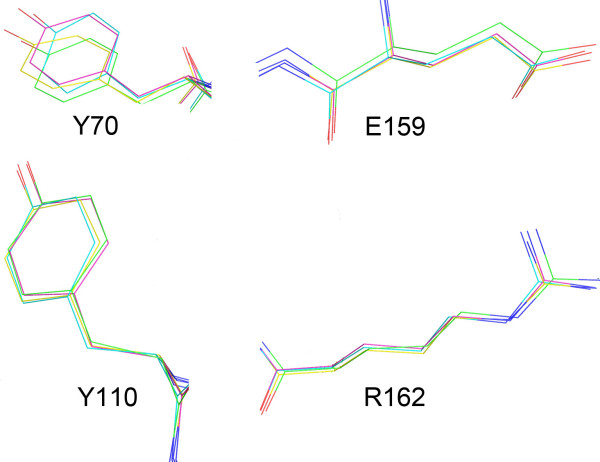
**Structural superposition of the active site residues**. Trichosanthin, α-momorcharin, β-luffin and luffaculin 1 are colored as green, cyan, magenta and yellow, respectively.

The active site is located in a cleft between the N-terminal domain and the C-terminal domain, which serves as the substrate-binding and catalysis site in RIPs (Fig. 1). Fig. 6 shows the conformations of luffaculin 1 catalytic residues, superimposed with those of other RIPs. The side chains of the active site residues have roughly the same position as the corresponding residues of other RIPs' structures [15,36], whereas Tyr70 shows relatively higher mobility in all analyzed RIPs. It has been reported that this residue interacts with the targeted adenine [37], and together with a second tyrosine (Tyr110 in luffaculin 1), forms an aromatic stack of π electron system. The conformational flexibility of Tyr70 side chain may promote the substrate recognition and the formation of the aromatic stack.

**Figure 7:**
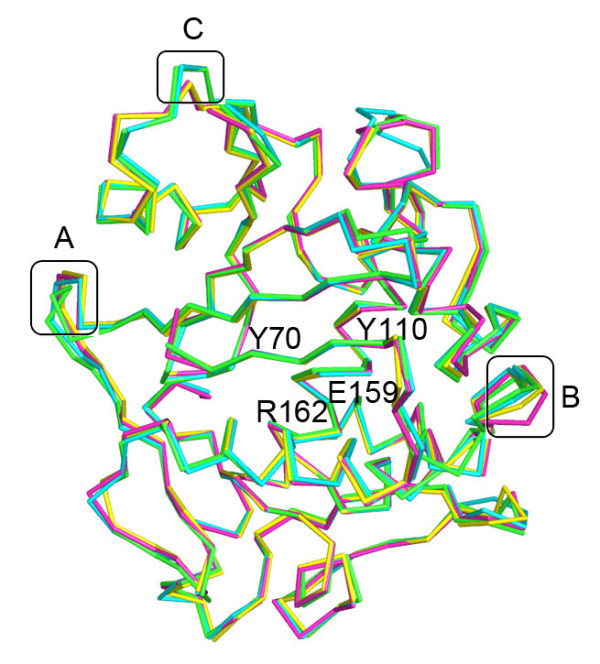
**Superposition of Cα atoms of trichosanthin (green), α-momorcharin (cyan), β-luffin (magenta) and luffaculin 1 (yellow)**. Loop deviations between β3-strand and α2-helix and between α8- and α9-helix are outlined in the boxes A and B, respectively. The largest deviation region at the β8-strand of the N-terminal region is outlined in the box C.

The final model of luffaculin 1 shows a common "RIP fold". The superposition of Cα atoms of luffaculin 1 to trichosanthin, α-momorcharin and β-luffin gave rmsd (root mean square deviation) values of 0.527, 0.492 and 0.359 Å, respectively. Despite this overall structural similarity of luffaculin 1 to other RIPs (Fig. 7), some noticeable differences exist in surface exposed loop regions particularly those between β3-strand and α2-helix, and between α8- and α9-helix. The largest deviation occurs at the β8-strand of the N-terminal region (box C in Fig. 7).

## Conclusion

We present the 1.4 Å resolution crystal structure of luffaculin 1 and its X-ray sequence. This sequence was derived based on the high resolution electron density, validated against the second molecule present in the crystals, the evaluation of the chemical environment of selected residues, and the sequence comparison with other homologues. A total of 86% (without using sequence comparison) or 94% (with sequence comparison) of luffaculin 1 residues can be assigned with confidence by this approach. The luffaculin 1 is quite similar to luffin a at both sequence and structural levels, suggesting its functions as an RIP.

## Methods

### Purification and crystallization

Luffaculin 1 was extracted and purified by extraction with acetate buffer, ammonium sulfate fractional precipitation and cation exchange chromatography [25]. It was eluted as a single symmetrical peak in a cation exchange column (Mono S, Amersham Pharmacia Biotech) and gave a single band with an apparent molecular weight of about 28 kDa by reducing SDS-PAGE. The purified luffaculin 1 was thoroughly dialyzed against deionized water and lyophilized. For crystallization, lyophilized powder of luffaculin 1 was dissolved to a concentration of 15.8 mg/mL and then crystallized by hanging drop vapor diffusion method [38] at room temperature by mixing 2 μL protein (15.8 mg/mL) with an equal volume of reservoir solution (28% (w/v) PEG 6000, 0.1 M citrate buffer pH4.5, containing 0.02% (w/v) sodium azide) and equilibrating against 800 μL of the same reservoir solution. The crystal was briefly dipped into a cryoprotectant, 20% of glycerol (final concentration) in the reservoir solution, before data collection.

### Data collection and processing

Diffraction data of the crystals were collected using synchrotron radiation (APS SER-CAT beamline 22ID) at low temperature (100K) to improve the diffraction quality and to decrease the radiation decay. A 1.4 Å resolution data set was obtained and the diffraction data were processed with the program package HKL2000 [39]. The crystals belong to space group P1, with unit-cell parameters a = 39.135 Å, b = 46.813 Å, c = 83.571 Å, α = 89.068°, β = 80.009°, γ = 72.143°. Matthews coefficient calculations [40] show two molecules present in the asymmetric unit and the value of Vm is 2.49 Å Da^-1 ^corresponding to a solvent content of 48%. The statistics for the data set are summarized in Table 1. The final merged data set of 94795 unique reflections has high quality with an Rmerge of 0.03 and an averaged signal to noise ratio of 21.8.

### Structure determination and model refinement

The structure of luffaculin 1 was solved by the molecular replacement method (AMORE [41,42]) using a homology model built based on the sequence of luffin a [43,44]. Luffin a and luffaculin 1 were both purified from the same genus but different species in the Cucurbitaceae family, and were expected to share high sequence homology and structure similarity. Rotational solutions in the resolution range of 8-4 Å showed two clear peaks with the high correlation coefficients of 0.223 and 0.199, respectively, and the corresponding third highest value was 0.071. Then, the translation vector of the first solution was fixed because of the space group P1 of luffaculin 1 and the translation search was performed for the second solution, giving a higher correlation coefficient of 0.429. The refinement was performed with the program CNS [33] in a resolution range from 50-1.4 Å. A total of 5% of the data was randomly selected for R_free _calculation throughout the whole refinement. After a starting cycle of rigid body refinement, the R factor was 0.3360 and R_free _was 0.3518. Simulated annealing and restrained individual B-factor refinement were then performed. The sigma A weighted 2Fo-Fc and Fo-Fc electron density maps were used to guide the model building process. The model was examined and manually rebuilt with the graphic program O [34]. In the final stage of the refinement, water molecules were added by CNS at locations where electron density was stronger than 3.0σ in sigma A-weighted Fo-Fc maps and had reasonable hydrogen-bond interaction with the protein, and then were inspected with program O. The carbohydrates and the polyethylene glycol (PEG) molecules [see Additional file 1] were visible at this stage in 2Fo-Fc and Fo-Fc electron density maps and were built in. Luffaculin 1 was crystallized from the solution containing PEG6000. It is inevitable that there are some low molecular weight polyethylene glycol molecules existed in the PEG6000 and these small molecules most likely penetrate into crystals. Such cases can also be found in the PDB data bank, for example, in PDB entry 2FD6 [45]. The final model has R factor and R_free _of 0.213 and 0.232, respectively, containing 492 water molecules, one PEG1 (tetraethylene glycol), two PEG2 (diethylene glycol) and four N-acetylglucosamines (NAG) in the asymmetric unit. Data collection and model refinement statistics are listed in Table 1.

## Authors' contributions

XMH refined the structure and drafted the manuscript. MHC obtained the initial crystallization conditions for luffaculin 1. EJM and LQC carried out the data collection and processing. MHC and MDH are advisors of XMH. MDH provided constructive advices and worked on the manuscript. JMX tested pharmacological activities of luffaculin 1. All authors read and approved the final manuscript.

## Supplementary Material

Additional File 1 **The electron density of one PEG1 and two PEG2**. These maps (2Fo-Fc composite omit maps) is contoured at 1σ. The format of the additional file is JPEG and the file can be viewed through ACD systems. Click here for file

## Figures and Tables

**Table 1:** Data collection and model refinement statistics for luffaculin 1

Space group	P1
Cell parameters	a = 39.135 Å, b = 46.813 Å, c = 83.571 Å, *α *= 89.068°, *β *= 80.009°, *γ *= 72.143°
Resolutions (Å)	1.4
Completeness (%)	86.7 (62.0)
Redundancy	1.9 (1.7)
Rmerge (%)^a^	0.03 (0.115)
Unique reflections	94795
I/σ(I)	21.8 (4.6)
Resolution range	50.00-1.40 (1.45-1.40)
R_work_	0.213 (0.250)
R_free_	0.232 (0.275)
No. of water molecules	492
No. of carbohydrates	4 per asymmetric unit
No. of polyethylene glycols	3
R.m.s.deviations from ideal geometry	
Bond lengths (Å)	0.007
Bond angles (°)	1.153

Values in parentheses refer to the highest resolution shell (1.45-1.40) Å.

^a ^Rmerge = Σ|*I*_i_-<I>|/Σ*I*_i_, where *I*_*i *_is the intensity of the *i*th observation and <*I*> is the mean intensity of the reflections.

**Table 2:** Assignment of the sequence of luffaculin 1 by X-ray sequencing method (total number of residues: 241)

	Sequence assignment method	Number of identified residues
1	Both molecule A and B have clear electron density and can be assigned unambiguously	151
		
2	Using method 1 plus electron density of the second molecule for those residues where the first molecule has weak electron density, 66% of residues (160) can be deduced	160 (9 additional residues were assigned: A123, A185, A206, A215, A219, A221, A222, A234 and B209)
		
3	Using methods 1 and 2 plus chemical environment evaluation without using sequence comparison, 86% of residues (208) can be deduced.	208 (exclude 28*, 29, 33*, 48*, 56*, 93*, 97, 109*, 111*, 122*, 132*, 142*, 156*, 157*, 169*, 177*,179, 190*, 197*, 202*, 204*, 205*, 213*, 216*, 217, 218, 220*, 223*, 225, 226*, 228, 237*, 241*)
		
4	Using method 3 plus the sequence comparison, 94% of residues (227) can be deduced.	227 (exclude 28*, 29, 97, 109**, 111**, 190*, 216*, 217, 218, 220*, 225, 228, 223*, 237*)
		
	Neither molecule A nor B has clear density map, but the sequence is highly conserved among RIPs	4 (A&B109*, A&B179, A&B111*, A&B223*)
		
	Neither molecule A nor B has clear density map, and the sequence is not conserved among RIPs	6 (A&B29, A&B97, A&B217, A&B218, A&B225, A&B228)

*These residues belong to residue pairs (Asn/Asp, Gln/Glu, Val/Thr) and cannot be distinguished by X-ray crystallography.

**These two residues are conserved, but the electron density of both molecules A and B isn't clear, so we cannot classify these two residues to Group 4.
