# Supplementary figures and images for: X-ray sequence and crystal structure of luffaculin 1, a novel type 1 ribosome-inactivating protein
Source: BMC Struct Biol. 2007 Apr 30;7:29. doi: 10.1186/1472-6807-7-29 (PMC1868734; doi:10.1186/1472-6807-7-29)

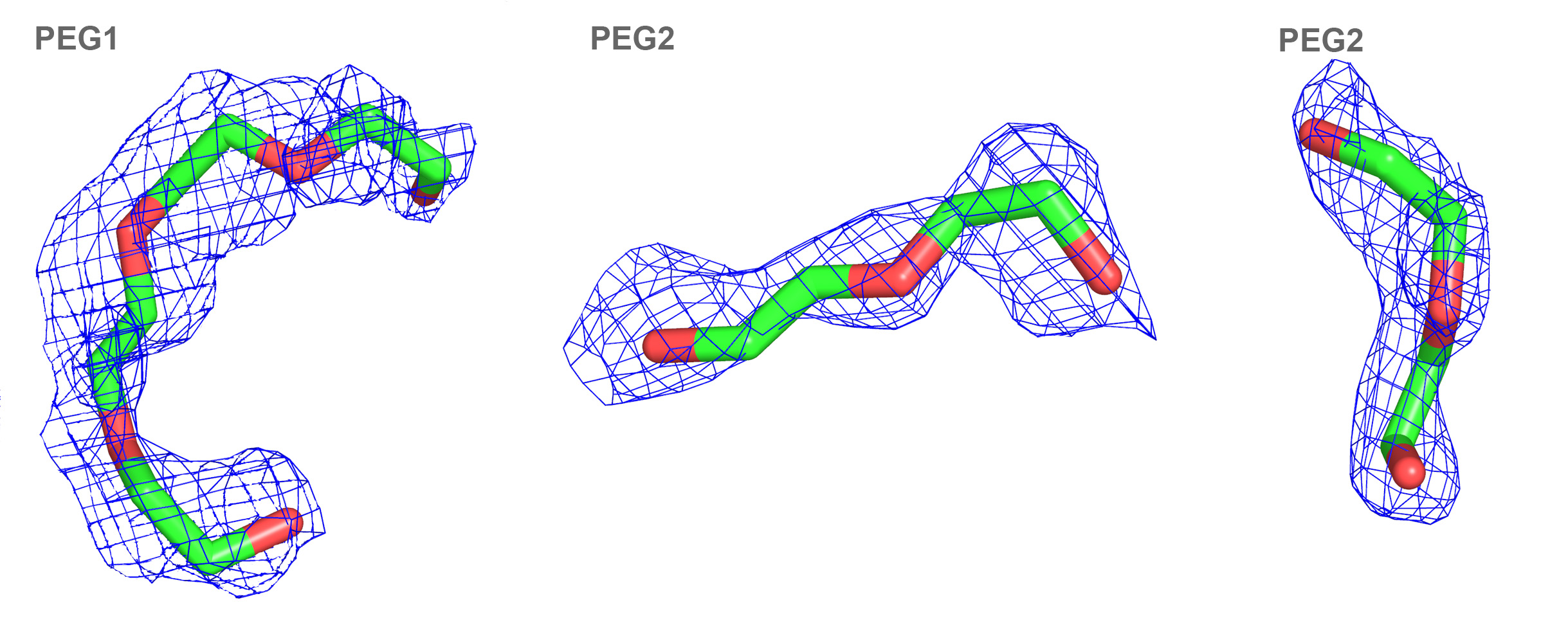

Supplement: Additional File 1 — The electron density of one PEG1 and two PEG2. These maps (2Fo-Fc composite omit maps) is contoured at 1σ. The format of the additional file is JPEG and the file can be viewed through ACD systems. [file 1472-6807-7-29-S1.jpeg]
